# Computational and Cellular Evaluation of the Anti-Melanogenic Potential of Gancidin W: Integrating Network Pharmacology and Free-Energy Analyses

**DOI:** 10.3390/ph19081191

**Published:** 2026-07-29

**Authors:** Yang Xu, Chang-Gu Hyun

**Affiliations:** Department of Chemistry and Cosmetics, Jeju National University, Jeju 63243, Republic of Korea; iamxuyang1990@gmail.com

**Keywords:** Gancidin W, melanogenesis, tyrosinase inhibition, diketopiperazine, depigmenting agent, molecular dynamics simulation, relative binding free-energy

## Abstract

**Background/Objectives:** Dysregulated melanogenesis underpins numerous hyperpigmentation disorders. Gancidin W, a naturally occurring diketopiperazine with diverse biological activities, has attracted interest as a potential bioactive compound; however, its role in melanogenesis remains largely unexplored. This study aimed to evaluate the anti-melanogenic potential of Gancidin W through an integrated experimental and computational approach. **Methods:** Network pharmacology analysis was performed to investigate the potential molecular mechanisms of Gancidin W against hyperpigmentation. The cellular effects of Gancidin W were evaluated in α-MSH-stimulated B16F10 melanoma cells by assessing intracellular tyrosinase activity, melanin content, and cytotoxicity. Furthermore, density functional theory (DFT) calculations, molecular dynamics (MD) simulations, MM/GBSA binding free-energy analysis, and relative binding free-energy (RBFE) calculations were conducted to investigate the molecular interactions of Gancidin W and its structurally related analogue Maculosin with human tyrosinase-related protein 1 (hTYRP1). **Results:** Network pharmacology analysis predicted potential targets and signaling pathways involved in melanogenesis regulation. Gancidin W reduced intracellular tyrosinase activity by 34.54% and melanin content by 25.09% without cytotoxic effects. Multiscale computational analyses demonstrated stable interactions of Gancidin W within the hTYRP1 active site and favorable binding energetics (Δ*G*_bind_ = −28.77 ± 0.75 kcal/mol). Compared with Maculosin, Gancidin W exhibited a more favorable binding profile, as supported by MM/GBSA and RBFE analyses, consistent with the inhibitory effects observed in the cellular assays. **Conclusions:** Gancidin W exhibits anti-melanogenic potential, and multiscale computational analyses provide molecular insights into its interaction with hTYRP1. These findings support further investigation of Gancidin W as a potential natural depigmenting agent for hyperpigmentation-related disorders.

## 1. Introduction

Diketopiperazines (DKPs) are the smallest cyclic dipeptide natural products formed via intramolecular cyclization of two α-amino acids, featuring a stable six-membered 2,5-diketopiperazine scaffold [1,2]. Owing to their conformational rigidity, structural diversity, and favorable physicochemical properties, DKPs have emerged as privileged scaffolds in natural product chemistry and drug discovery [3,4]. Compared with linear peptides, their cyclic architecture enhances metabolic stability, enzymatic resistance, and membrane permeability, while also enabling diverse non-covalent interactions with biological macromolecules [1,5,6,7].

DKPs are widely distributed across bacteria, fungi, plants, and marine-derived microorganisms, with marine environments serving as a particularly rich source of structurally diverse analogues [8,9,10]. Environmental pressure in marine ecosystems drives the evolution of specialized secondary metabolism, leading to DKPs with extensive structural diversification [11]. These include prenylated, halogenated, sulfur-bridged, and fused-ring derivatives, which collectively underpin their broad biological activities [1,12,13].

From a biosynthetic perspective, DKPs are mainly generated through non-ribosomal peptide synthetase (NRPS)-dependent and cyclodipeptide synthase (CDPS)-mediated pathways [14,15,16]. NRPS systems enable incorporation of non-proteinogenic amino acids and extensive tailoring modifications, whereas CDPS pathways directly assemble cyclic dipeptides from aminoacyl-tRNAs followed by post-modifications such as oxidation, methylation, and prenylation [17,18]. Together, these pathways contribute to the remarkable chemical diversity and functional plasticity of DKPs.

Accumulating evidence indicates that DKPs exhibit diverse biological activities, including antibacterial, antifungal, antiviral, anti-inflammatory, antioxidant, anticancer, neuroprotective, and immunomodulatory effects [13,19,20,21]. Beyond classical pharmacological roles, DKPs have also been implicated as small-molecule mediators in microbial communication systems, including quorum sensing and biofilm regulation [22,23,24], suggesting that they may participate in ecological signaling and adaptive regulation processes [25].

Cyclo(*L*-Leu-*L*-Pro), also known as Gancidin W, is a representative DKP natural product originally isolated from *Streptomyces gancidicus* [26]. It is widely distributed across diverse microorganisms, including *Streptomyces*, *Bacillus*, *Pseudomonas*, *Lactobacillus*, and marine-derived fungi [27,28,29,30,31], and has also been detected in plants, fermented foods, and animal-associated systems [32,33,34,35], indicating broad ecological prevalence. Functionally, Gancidin W exhibits multiple biological activities, including antimicrobial, antifungal, antioxidant, anti-inflammatory, and anticancer effects [36,37,38,39,40]. Importantly, its free radical scavenging and cytoprotective properties have been documented [41,42], suggesting potential relevance to oxidative stress-associated biological processes. Furthermore, preliminary evidence has demonstrated that Gancidin W can inhibit mushroom tyrosinase (mTYR) activity [43]. Collectively, these findings indicate that Gancidin W may represent a promising candidate for the regulation of melanogenesis. However, its effects on mammalian melanogenesis and the underlying molecular mechanisms remain largely unexplored.

Melanogenesis is a tightly regulated biological process controlled primarily by tyrosinase (TYR), the rate-limiting enzyme responsible for melanin biosynthesis [44]. Dysregulation of this pathway leads to hyperpigmentation disorders such as melasma and post-inflammatory hyperpigmentation [45,46]. Although clinically used TYR inhibitors such as kojic acid and hydroquinone are available, their application is limited by instability, cytotoxicity, and safety concerns [47]. Therefore, the identification of structurally novel and mechanistically well-characterized TYR inhibitors remains a critical need in cosmeceutical and pharmaceutical development.

**Figure 1:**
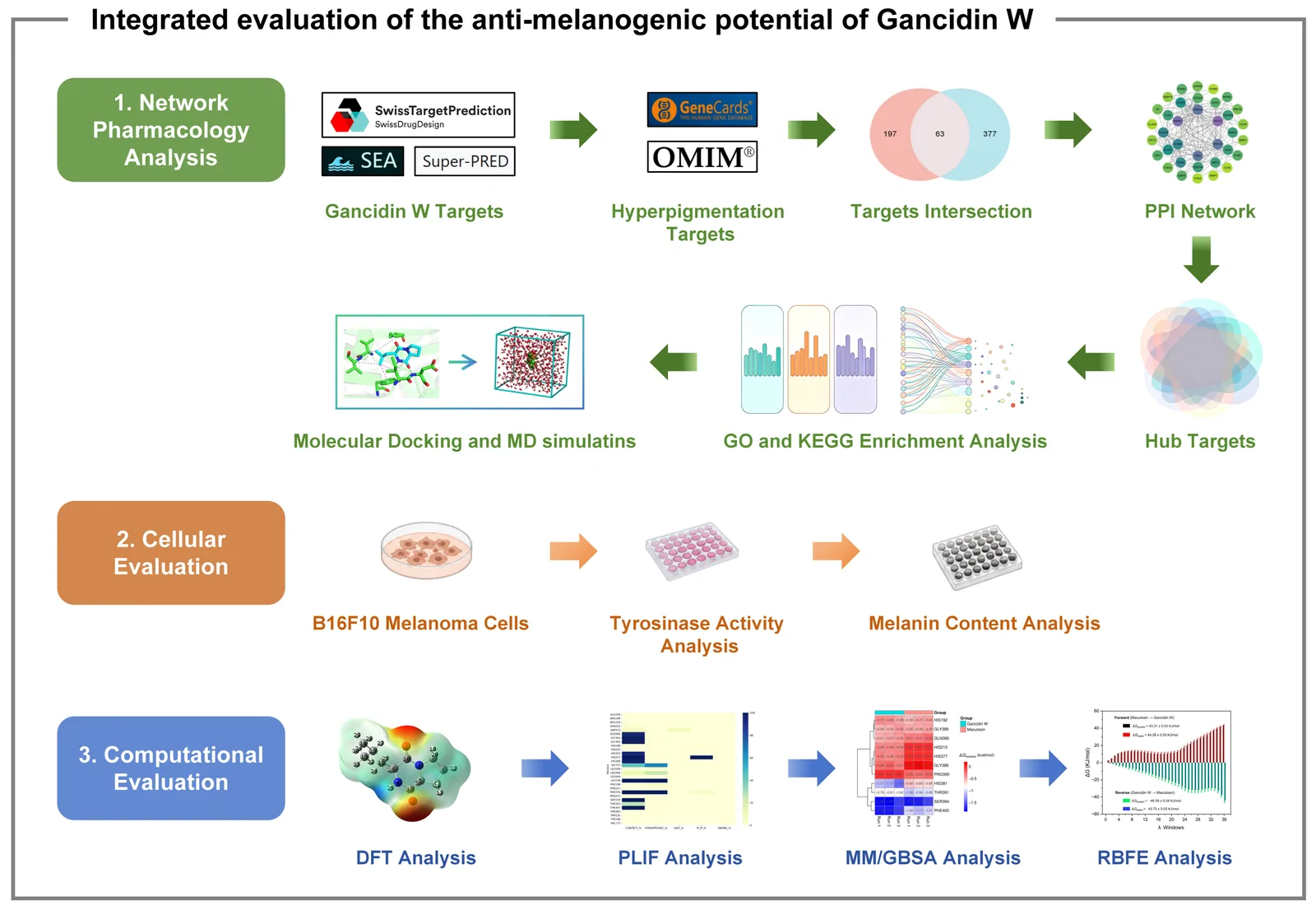
Schematic illustration of the overall study workflow.

To evaluate the anti-melanogenic potential of Gancidin W, an integrated experimental and computational strategy was employed. Network pharmacology was initially used to explore the relevance of Gancidin W to melanogenesis-related targets, providing a rationale for subsequent evaluation of melanin production and intracellular tyrosinase activity. Building upon these findings, a multiscale computational framework integrating DFT calculations, molecular docking, molecular dynamics simulations, MM/GBSA analyses, and RBFE calculations was established to characterize the structural basis, interaction network, and thermodynamic drivers underlying the binding of Gancidin W to human tyrosinase-related protein 1 (hTYRP1). A schematic overview of the integrated workflow adopted in this study is presented in Figure 1. Collectively, this study not only evaluates the anti-melanogenic potential of Gancidin W but also provides molecular-level insights into its recognition and stabilization within the hTYRP1 binding site, offering a theoretical foundation for the future development of diketopiperazine-derived anti-melanogenic agents.

## 2. Results and Discussion

### 2.1. Network Pharmacology Analysis of Gancidin W A in Melanogenesis

#### 2.1.1. Identification of Targets Related to the Anti-Melanogenic Effect of Gancidin W

**Figure 2:**
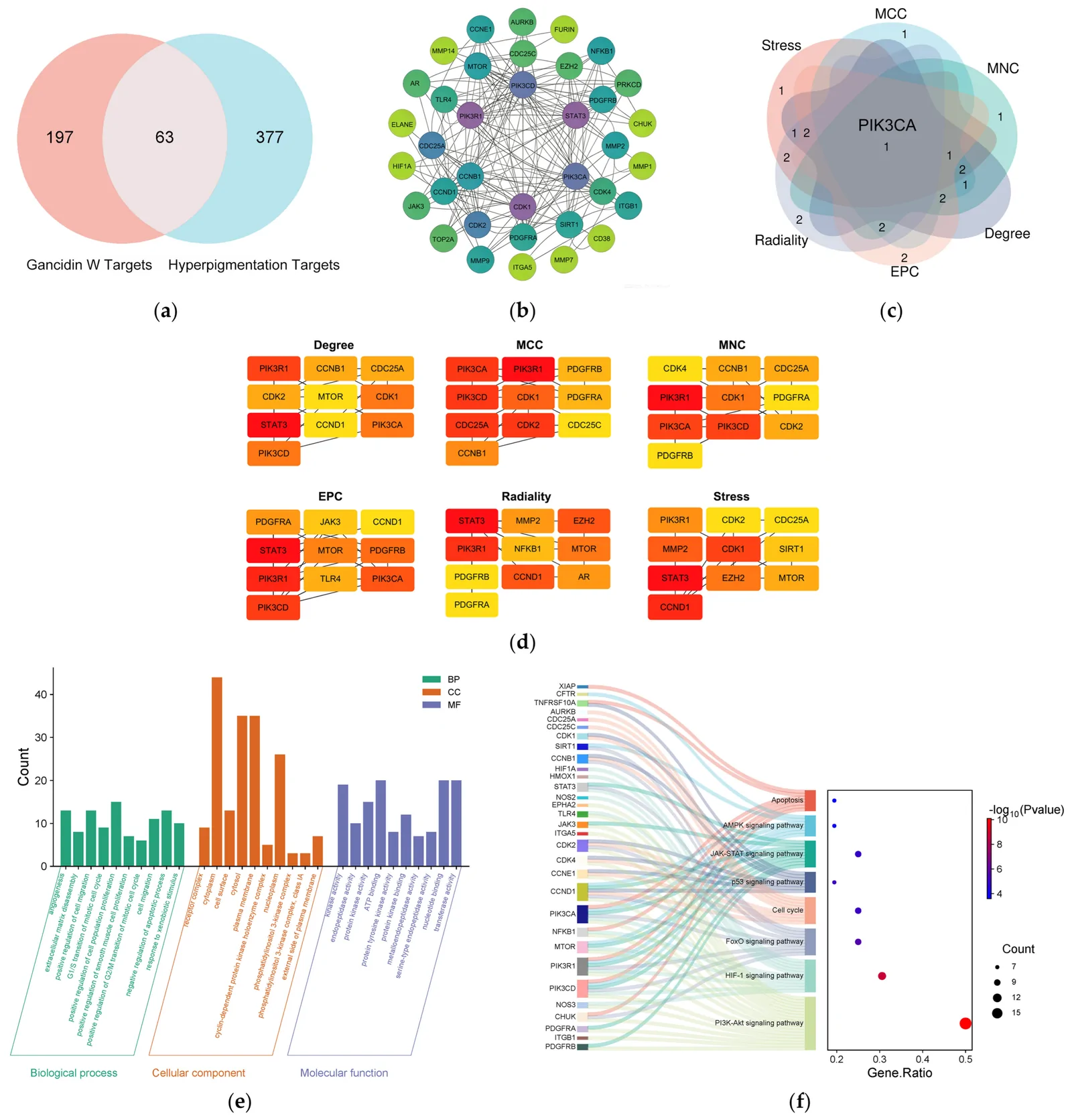
Identification of potential therapeutic targets of the microbiota metabolite Gancidin W against hyperpigmentation disorders: (**a**) Overlapping targets shared by Gancidin W targets and hyperpigmentation-related targets. (**b**) PPI network constructed for the overlapping targets. (**c**) Identification of core targets using six topological algorithms visualized by a Venn diagram. (**d**) Hub target screening using six algorithms implemented in the CytoHubba plug-in of Cytoscape. (**e**) Gene Ontology (GO) enrichment analysis showing the top 10 significantly enriched terms in biological process (BP), cellular component (CC), and molecular function (MF) based on *p*-values. (**f**) Kyoto Encyclopedia of Genes and Genomes (KEGG) pathway enrichment analysis showing the top 10 significantly enriched pathways ranked by *p*-value.

A total of 138, 35, and 177 potential targets of Gancidin W were obtained from the STP, SEA, and SuperPred databases, whereas 496 and 334 hyperpigmentation-related targets were retrieved from the GeneCards and OMIM databases, respectively. Following integration, deduplication, and HGNC nomenclature standardization, 260 Gancidin W-related targets and 440 hyperpigmentation-related targets were retained. Intersection analysis identified 63 common targets (Figure 2a, Table S1), which were predicted as potential targets associated with Gancidin W and hyperpigmentation-related processes and were subjected to subsequent network pharmacology analyses.

#### 2.1.2. Identification of Core Targets in the PPI Network

The PPI network comprised 36 nodes and 164 edges, illustrating the interaction landscape of the overlapping targets associated with hyperpigmentation and providing insights into the potential pharmacological actions of Gancidin W (Figure 2b). To ensure the robustness of hub target identification, six topological metrics from the cytoHubba plugin (Degree, MCC, MNC, EPC, Stress, and Radiality) were employed to rank candidate targets in the PPI network. The top 10 targets identified by each algorithm were further subjected to Venn intersection analysis (Figure 2d). Among these candidates, PIK3CA was identified as the only overlapping target shared by all six algorithms (Figure 2c), suggesting that it may serve as a key hub mediating the pharmacological effects of Gancidin W against hyperpigmentation.

Interestingly, functional inspection of the hub target distribution revealed the presence of two potential signaling-associated modules within the PPI network. The first module was characterized by a PI3K-centered signaling network comprising PIK3CA, PIK3CD, MTOR, and STAT3, highlighting the potential involvement of this signaling axis in cellular survival and melanogenesis regulation [48]. The second module was characterized by a cluster of cell cycle regulators, including CDK1, CDK2, CCND1, CDC25A, and CCNB1, highlighting a potential link between cell-cycle progression and melanocyte function [49,50]. These findings suggest that Gancidin W may regulate hyperpigmentation through multiple interconnected signaling mechanisms rather than a single pathway, providing a systems-level understanding of its predicted mode of action.

#### 2.1.3. GO Functional Annotation and KEGG Pathway Enrichment

Based on the 36 core targets identified from the PPI network, GO enrichment analysis revealed that these genes were primarily associated with cell-cycle regulation, cell proliferation, apoptosis, cellular migration, and response to xenobiotic stimulus (Figure 2e). Notably, the enrichment of terms related to the G1/S and G2/M transitions was consistent with the cell cycle-associated module identified in the PPI network, which was potentially driven by key regulators such as CDK1, CDK2, CCND1, and CDC25A [51,52].

Cellular component analysis indicated that the enriched targets were mainly localized in the cytoplasm, plasma membrane, receptor complexes, and the phosphatidylinositol 3-kinase complex. Molecular function analysis showed significant enrichment in kinase activity, protein kinase activity, ATP binding, and protein kinase binding, implicating kinase-mediated signaling as a potential contributor to the biological effects of Gancidin W as predicted in silico [53].

KEGG pathway analysis identified several significantly enriched pathways, including PI3K–Akt, HIF-1, FoxO, JAK–STAT, p53, and cell cycle signaling pathways (Figure 2f). Among these, the PI3K–Akt pathway involved multiple recurrent hub targets, including PIK3CA, PIK3CD, PIK3R1, MTOR, and STAT3, highlighting a potential PI3K-centered regulatory module within the interactome [48].

The enrichment of HIF-1 and FoxO signaling pathways further suggests potential associations with cellular stress response and survival-related processes [54]. In addition, p53 and cell cycle pathways were also significantly enriched, suggesting that the predicted targets may be involved in regulatory processes associated with melanogenesis and cellular homeostasis [55,56].

Collectively, the network pharmacology analyses provide a systems-level overview of the predicted biological functions and potential molecular mechanisms associated with the pharmacological effects of Gancidin W against hyperpigmentation. These findings provide a rational basis for future mechanistic studies but require further experimental validation.

#### 2.1.4. Molecular Docking Analysis of Core Hub Targets

**Figure 3:**
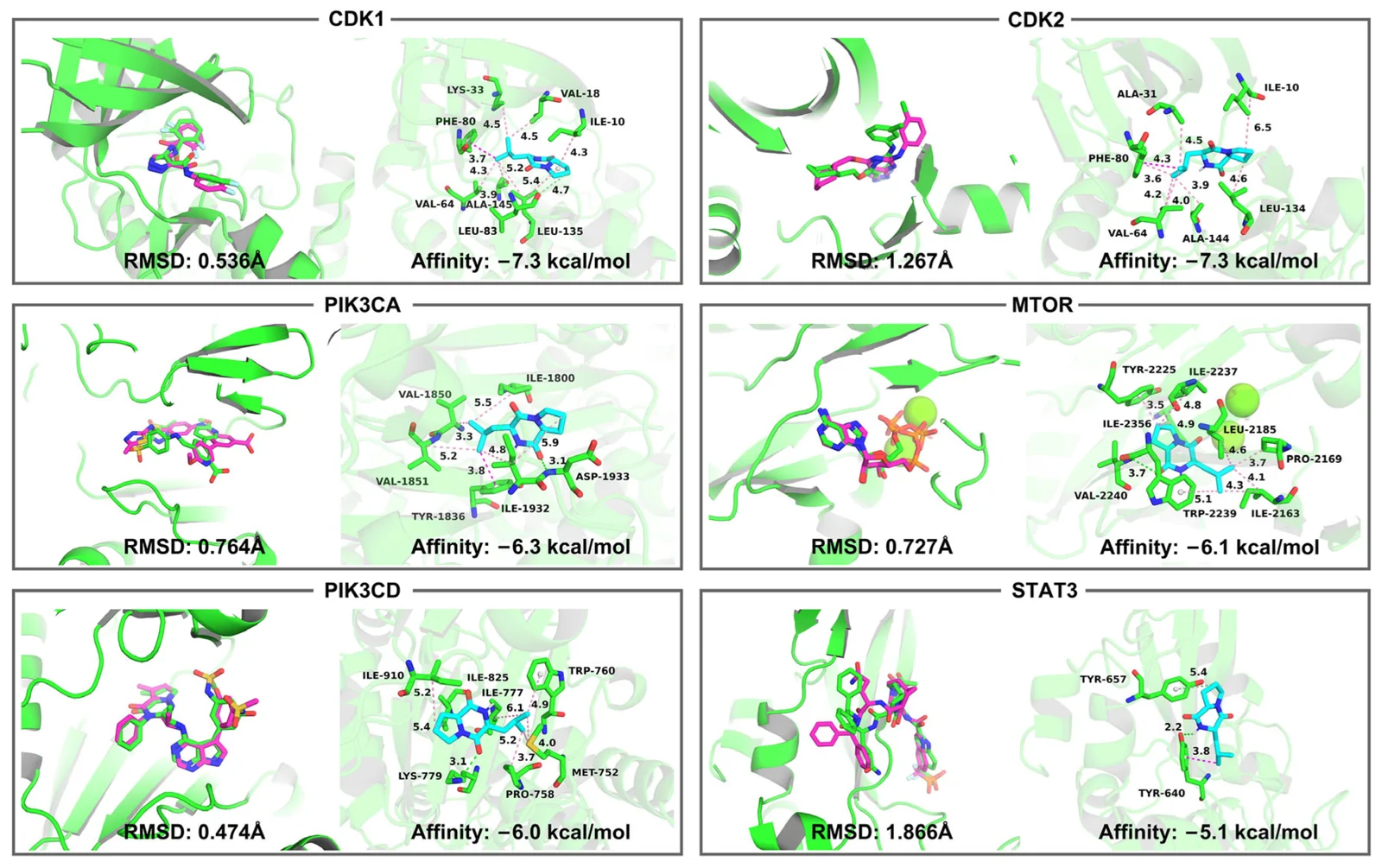
Molecular docking analysis of Gancidin W and methodology validation across six core hub targets. (**Left panels**) Superimposition of co-crystallized ligands (green) and re-docked conformations (purple) for methodological RMSD verification. (**Right panels**) 3D binding poses and detailed intermolecular interaction networks of Gancidin W within the active sites of the respective targets.

Molecular docking was conducted to investigate the interactive capacity of Gancidin W with the core network hubs. Guided by a comprehensive topological analysis using Degree centrality as the primary metric, six representative targets with structurally resolved ligand-binding domains were systematically prioritized: CDK1, CDK2, PIK3CA, PIK3CD, MTOR, and STAT3. Other prominent nodes (including CDC25A, PIK3R1, CCNB1, and CCND1) were excluded from further simulation, owing to the lack of well-defined small-molecule binding sites or their primary structural roles as scaffold/regulatory proteins. Prior to evaluating the target compound, the reliability of the docking procedure was confirmed by re-docking the native co-crystallized ligands. The resulting redocked conformations closely replicated the experimental binding modes, with RMSD values consistently <2.0 Å (Figure 3, left panels).

Gancidin W yielded favorable binding free energies across all six targets, ranging from −5.1 to −7.3 kcal/mol (Figure 3, right panels). The highest predicted affinities were obtained for CDK1, CDK2, and PIK3CA, with scores of −7.3, −7.3, and −6.3 kcal/mol, respectively. Docking analysis indicated that the binding poses were sustained primarily by conventional hydrogen bonds (green dashed lines), carbon–hydrogen bonds (light green dashed lines), and π-associated interactions (purple and pink dashed lines). These initial computational profiles suggest that Gancidin W possesses the structural compatibility to fit into the binding pockets of these core targets.

#### 2.1.5. MD Simulation Analysis of Gancidin W–Target Complexes

To assess the dynamic stability of the docking-derived binding modes, 100 ns MD simulations were performed for the Gancidin W-bound complexes (with CDK1, CDK2, and PIK3CA) alongside their respective co-crystallized reference systems. The RMSD profiles of all three complexes converged rapidly and remained stable throughout the trajectories, exhibiting fluctuations comparable to those of the reference systems (Figure S1). This structural stability was further supported by RMSF, radius of gyration (Rg), and solvent-accessible surface area (SASA) profiles. Additionally, hydrogen bond analysis confirmed the persistent maintenance of at least one intermolecular hydrogen bond within each complex under dynamic conditions, corroborating the structural integrity of the initial docking poses.

**Figure 4:**
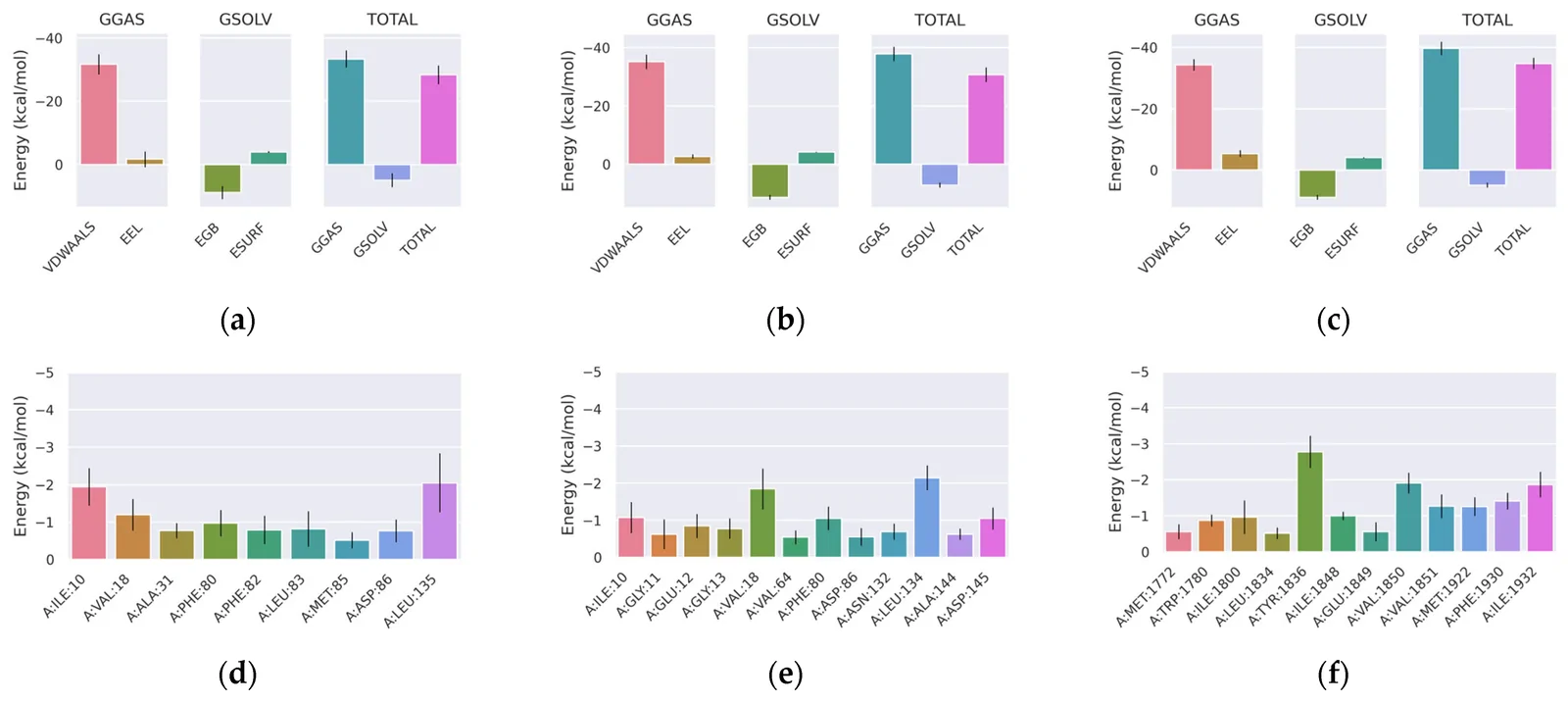
MM/GBSA binding free energies and per-residue energy decomposition of Gancidin W–target complexes. The MM/GBSA energy components, including van der Waals (Δ*E*_vdW_, VDWAALS), electrostatic (Δ*E*_ele_, EEL), polar solvation (Δ*G*_polar_, EGB), non-polar solvation (Δ*G*_nonpolar_, ESURF), gas-phase free energy (Δ*E*_gas_, GGAS), solvation free energy (Δ*G*_solv_, GSOLV), and total binding free energy (Δ*G*_bind_, TOTAL), are presented for (**a**) CDK1, (**b**) CDK2, and (**c**) PIK3CA; the corresponding per-residue energy decomposition profiles identifying key hotspot residues are shown for (**d**) CDK1, (**e**) CDK2, and (**f**) PIK3CA.

To evaluate the thermodynamic stability and energetic contributions governing the binding of Gancidin W to CDK1, CDK2, and PIK3CA, end-state binding free energies (Δ*G*_bind_) were calculated via the MM/GBSA method. Gancidin W exhibited robust affinities across all three systems, yielding Δ*G*_bind_ values of −34.80, −28.39, and −30.75 kcal/mol for CDK1, CDK2, and PIK3CA, respectively (Figure 4a–c, Tables S2–S4). Deconstruction of the total binding free energy revealed that van der Waals (Δ*E*_vdw_) interactions dominated the overall complex stabilization.

Per-residue energy decomposition was subsequently performed to identify the predominant residues governing the molecular recognition (Figure 4d–f, Tables S5–S7). For the CDK1 complex, ILE10 and LEU135 emerged as the primary energetic contributors. In the CDK2 system, the major interaction energy was localized at residues ILE10, VAL18, and LEU134. For the PIK3CA complex, the binding was primarily sustained by the significant energy contributions of TYR1836, VAL1850, and ILE1932. These thermodynamic profiles provided explicit energetic evidence that cross-validated the binding modes predicted by the initial molecular docking.

### 2.2. Cellular Evaluation of the Anti-Melanogenic Activity of Gancidin W

Computational intersection analysis identified Gancidin W as a potential regulator of melanogenesis-associated genes. To validate this, we quantified intracellular tyrosinase activity and melanin content as key hallmarks of melanogenesis in B16F10 melanoma cells. Given our prior evidence that Gancidin W directly inhibits mTYR, we assessed these cellular parameters to confirm the inhibitory effect on melanin biosynthesis.

**Figure 5:**
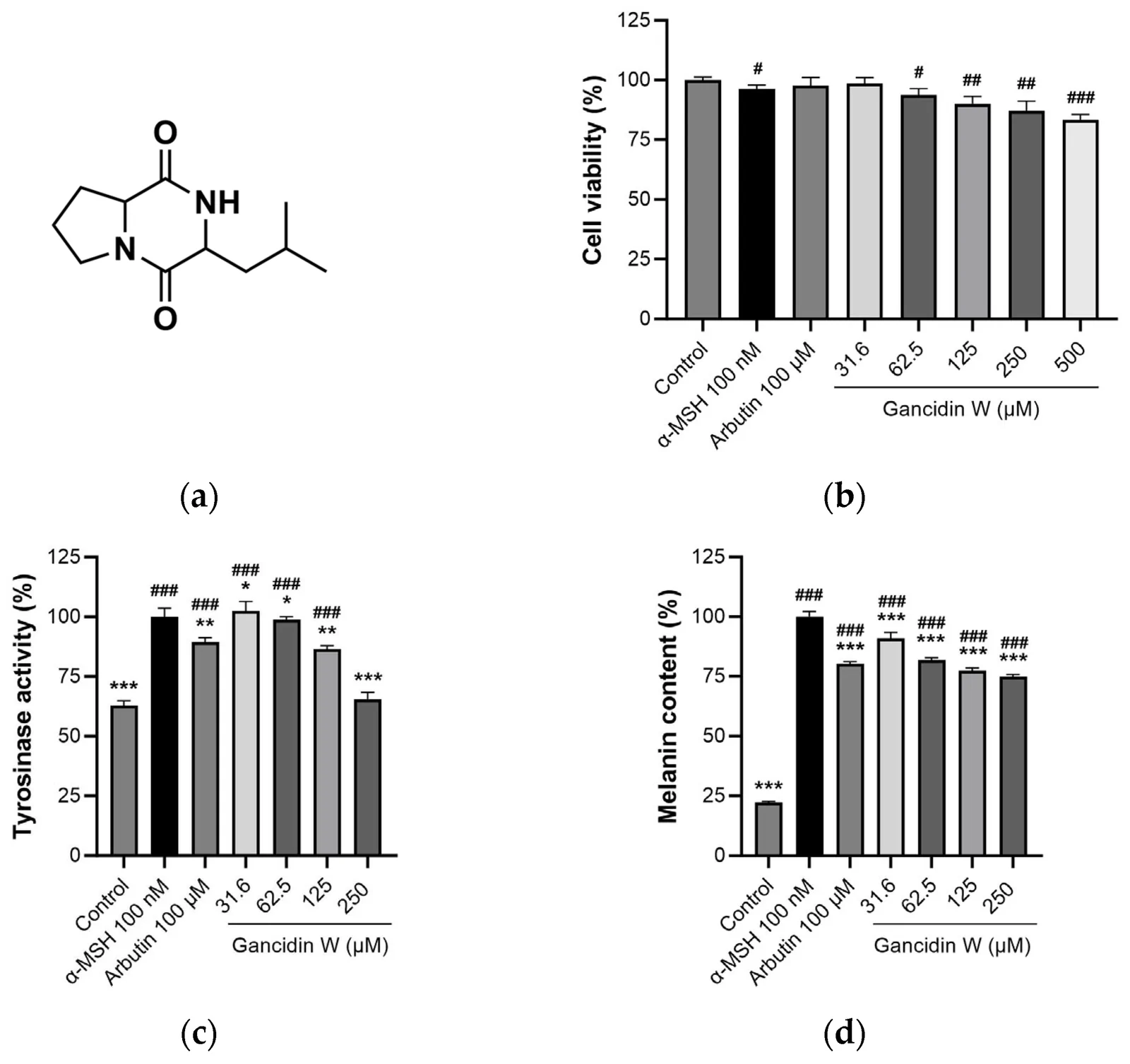
Inhibitory effects of Gancidin W on tyrosinase activity and melanogenesis in B16F10 cells: (**a**) Chemical structure of Gancidin W. (**b**) Cell viability quantified by MTT assay. (**c**) Intracellular tyrosinase activity and (**d**) melanin content in α-MSH-stimulated B16F10 cells. Arbutin was used as the positive control. All the experiments were conducted after 72 h of treatment. Data are presented as mean ± SD (*n* = 3). ^#^ *p* < 0.05, ^##^ *p* < 0.01, ^###^ *p* < 0.001 vs. untreated control; * *p* < 0.05, ** *p* < 0.01, *** *p* < 0.001 vs. α-MSH-treated group.

Prior to functional assays, the cytotoxicity of Gancidin W was evaluated by the MTT assay. Cell viability remained at 90.43% following treatment with 250 μM Gancidin W, whereas 500 μM reduced cell viability to 86.65% (Figure 5b). Therefore, concentrations up to 250 μM were used for the subsequent intracellular tyrosinase activity and melanin content assays. At 250 μM, Gancidin W significantly reduced intracellular tyrosinase activity by 34.54% and melanin accumulation by 25.09% in α-MSH-stimulated B16F10 cells (Figure 5c,d).

Collectively, these findings demonstrate that Gancidin W possesses anti-melanogenic activity in vitro without compromising cell viability. Nevertheless, further studies using primary human melanocytes and reconstructed human skin models are needed to validate these findings and assess their relevance to human skin.

### 2.3. Multiscale Computational Analysis of Gancidin W and Maculosin

#### 2.3.1. Density Functional Theory (DFT) Analysis

**Figure 6:**
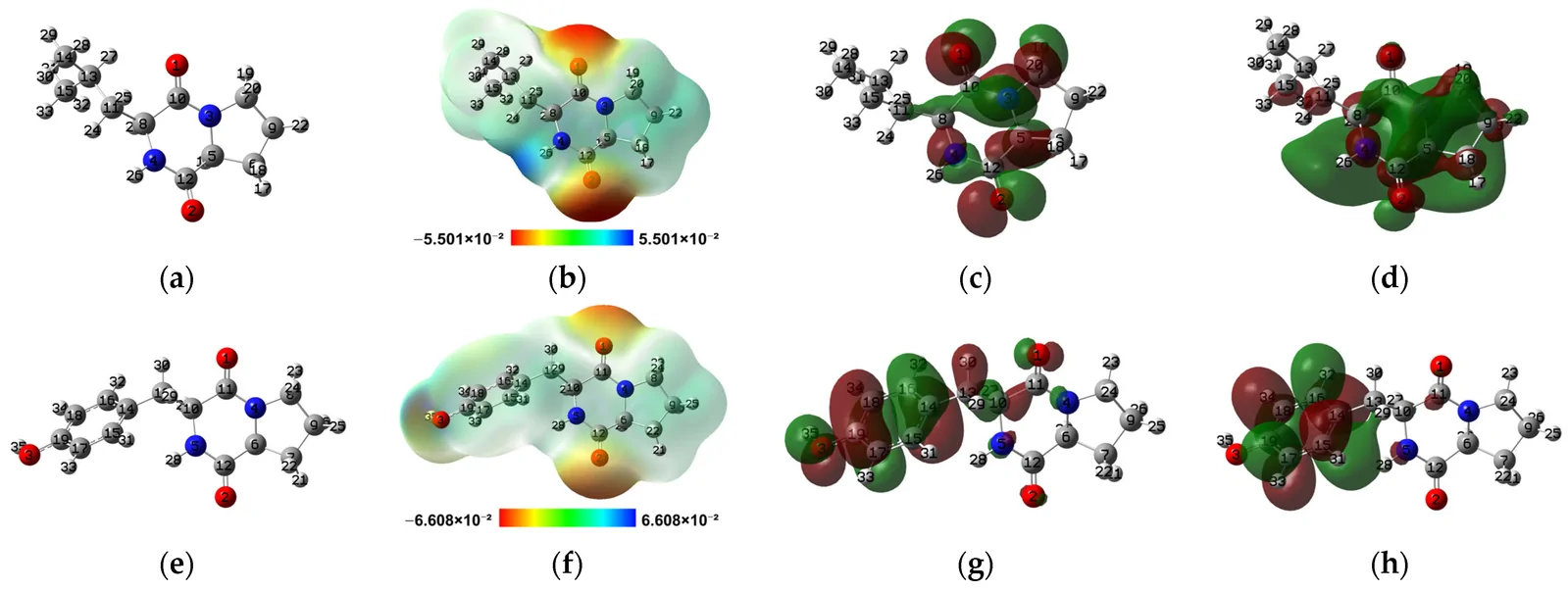
DFT-calculated electronic properties of Gancidin W (**a**–**d**) and Maculosin (**e**–**h**): (**a**,**e**) Optimized structures; (**b**,**f**) MEP surfaces; (**c**,**g**) HOMO distributions; and (**d**,**h**) LUMO distributions.

To investigate the substituent effects on the electronic structures of cyclic dipeptides, DFT calculations were performed on Gancidin W and Maculosin, a structurally related analogue exhibiting similar biological activities [57]. FMO analysis revealed distinct differences in the spatial localization of the frontier molecular orbitals between the two compounds [58] (Figure 6). For Gancidin W, both the HOMO and LUMO were found to be localized primarily on the DKP scaffold, with eigenenergies of *E*_HOMO_ = −6.84 eV and *E*_LUMO_ = −0.37 eV, yielding a HOMO–LUMO gap (Δ*E*_gap_) of 6.48 eV. In contrast, the FMOs of Maculosin shifted toward the *p*-hydroxyphenyl side chain, with diminished orbital densities on the DKP scaffold. Concurrently, Maculosin exhibited altered energy levels (*E*_HOMO_ = −6.48 eV and *E*_LUMO_ = −0.88 eV), resulting in a narrower Δ*E*_gap_ of 5.60 eV. These computational results indicated that the aromatic side chain modifies the electronic properties of the DKP core.

To assess the influence of substituents on the electrostatic topology, molecular electrostatic potential (MEP) mapping was conducted [59]. The computed MEP of Gancidin W was characterized by alternating regions of negative potential near the carbonyl oxygen atoms and positive potential across the amide fragments of the DKP ring. Conversely, while Maculosin maintained the fundamental MEP features of the DKP scaffold, an expanded region of negative potential was observed over the aromatic ring. This electronic redistribution is attributed to the electron-donating nature of the phenolic hydroxyl group, which induced a localized increase in negative electrostatic potential over the aromatic region.

Finally, the calculated dipole moments further quantify the divergence in the overall electronic profiles. The dipole moments of Gancidin W and Maculosin were calculated as 1.80 D and 2.63 D, respectively. The higher dipole moment observed for Maculosin signified a greater degree of electronic asymmetry, consistent with the enhanced molecular polarization induced by the aromatic side chain.

#### 2.3.2. Protein–Ligand Interaction and Binding Mode Analysis

**Figure 7:**
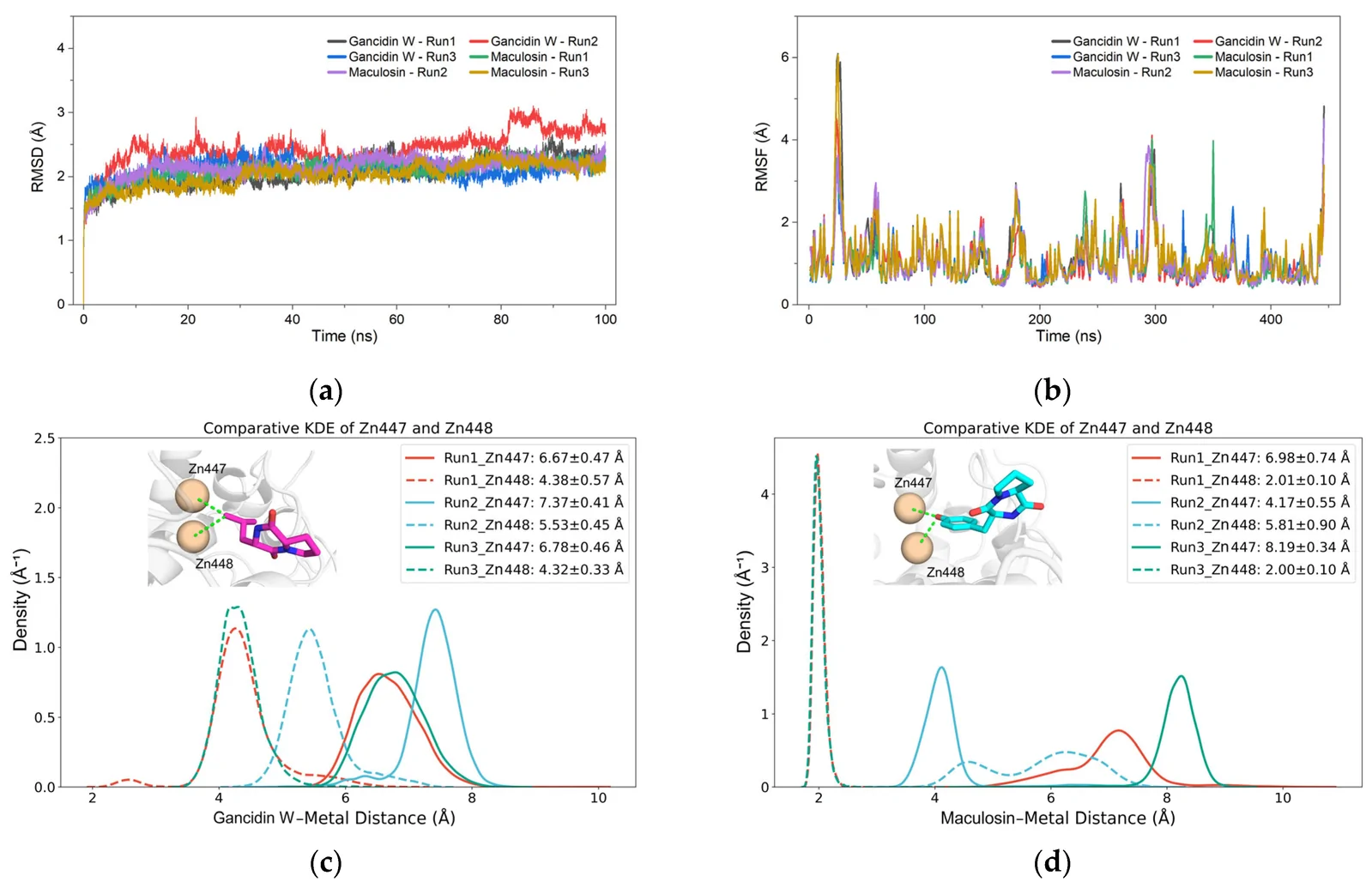

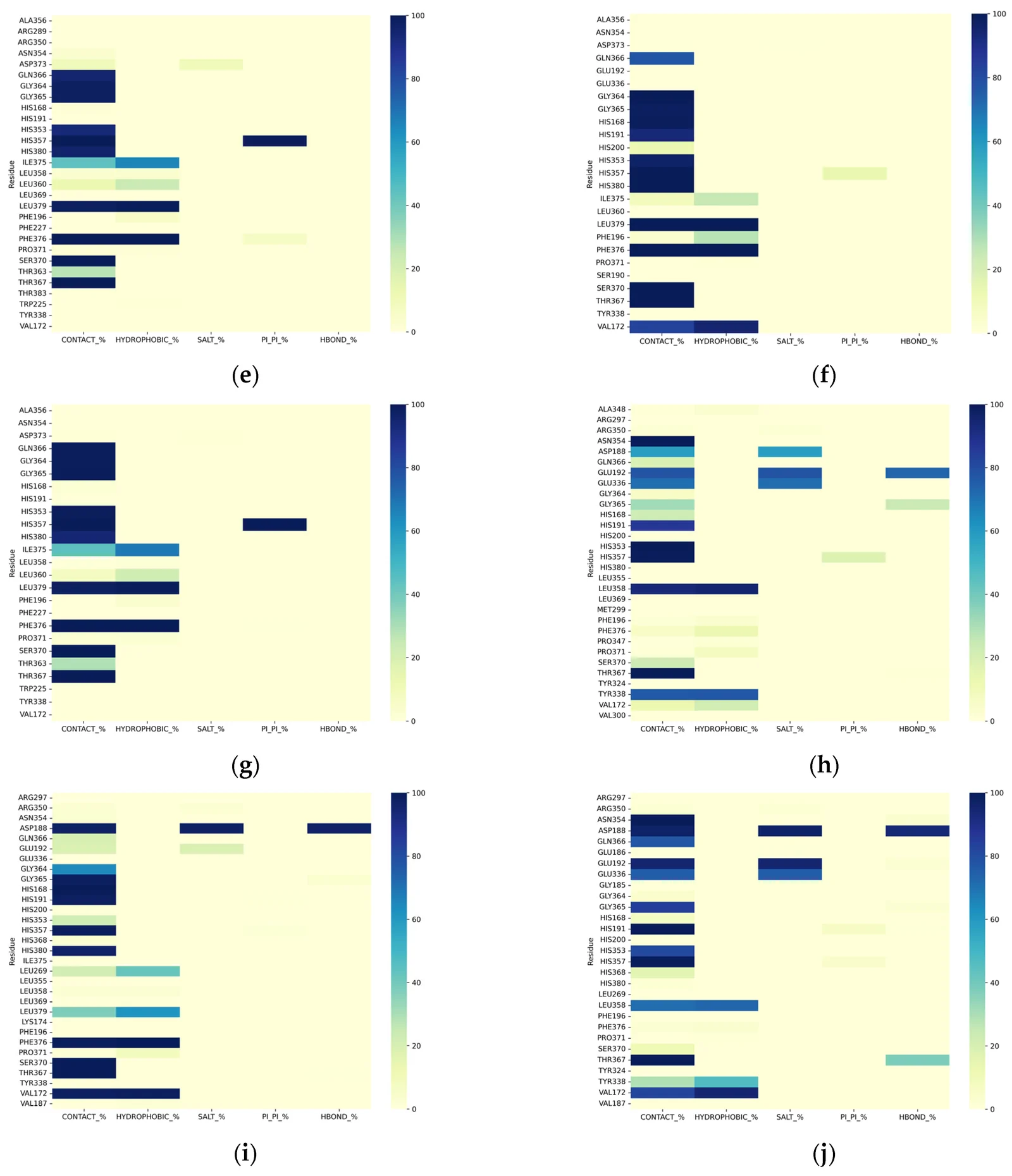
Binding landscape and interaction analysis of Gancidin W and Maculosin: (**a**) RMSD profiles; (**b**) RMSF profiles; (**c**) KDE distribution: Gancidin W; (**d**) KDE distribution: Maculosin; (**e**–**g**) PLIF profiles: Gancidin W (Runs 1–3); (**h**–**j**) PLIF profiles: Maculosin (Runs 1–3).

In the absence of an experimentally resolved hTYR crystal structure, hTYRP1 was utilized as a structural template due to its high homology (~40% identity) and a conserved binuclear active site that resembles hTYR [44]. Across all the independent trajectories, the comparable convergence behaviors observed in both RMSD and RMSF profiles confirm the structural stability of the systems and the high reproducibility of the statistical sampling (Figure 7a,b).

KDE analysis reveals distinct binding landscapes for the two DKP-based ligands, correlated with their side-chain functionalities (Figure 7c,d). Gancidin W, featuring an isopropyl group, exhibits a broad distance distribution (4.3–7.4 Å) with low probability density (<1.5 Å^−1^), indicating an absence of localized coordination. In contrast, Maculosin displays a bimodal distribution; notably, in two of the three trajectories, a dominant population emerges at ~2.0 Å (peak density ≈ 4.5 Å^−1^). The peak at ~2.0 Å aligns with typical Zn–O/N bond lengths [60]. Given that coordination geometry influences Zn-center affinity [61], the high occupancy at ~2.0 Å suggests that the *p*-hydroxyphenyl moiety facilitates stable coordination, increasing the ligand’s residence time within the Zn coordination sphere.

PLIF analysis based on the triplicate MD trajectories reveals significant divergence in the thermodynamic driving forces governing the binding of Maculosin and Gancidin W (Figure 7e–j). Maculosin exhibits a “recognition-oriented” binding mode, characterized by a robust electrostatic network centered on ASP188 and GLU192 (comprising hydrogen bonds and salt bridges). This polar network, coupled with hydrophobic sub-pockets defined by VAL172, LEU358, and PHE376, effectively anchors the ligand within the binding site. In contrast, Gancidin W displays an “occupancy-based” binding mode, where binding stability is predominantly driven by extensive hydrophobic contacts with residues such as PHE376, LEU379, and GLN366. Although Gancidin W displays significant contact populations in the vicinity of GLY364/365 and the histidine cluster (HIS353/380), PLIF analysis suggests that these interactions are presumably driven by steric and van der Waals contributions, arising from geometric complementarity rather than specific molecular recognition motifs.

In summary, Maculosin and Gancidin W adopt distinct binding strategies characterized by electrostatic anchoring and hydrophobic occupancy, respectively. These findings provide a theoretical framework for understanding the structure-activity relationship (SAR) of these two ligands.

#### 2.3.3. MM/GBSA Binding Free Energy and Per-Residue Energy Decomposition

Based on the equilibrated MD trajectories, MM/GBSA calculations were performed to estimate the energetic contributions to ligand binding (Table 1, Table S8). Gancidin W exhibited a slightly more favorable binding energy than Maculosin (−28.77 ± 0.75 vs. −24.32 ± 0.57 kcal/mol). Energy decomposition analysis revealed distinct energetic features underlying their binding behaviors. Gancidin W displayed a more favorable van der Waals contribution (Δ*E*_vdw_ = −32.51 kcal/mol), indicating that its binding is primarily driven by extensive hydrophobic contacts and geometric complementarity, consistent with the hydrophobic occupancy mode identified by PLIF analysis. In contrast, Maculosin showed markedly stronger electrostatic interactions (Δ*E*_ele_ = −54.44 kcal/mol), in agreement with its stable polar interaction network involving ASP188 and GLU192, as well as its propensity to maintain Zn^2+^ coordination during the simulations. However, the enhanced electrostatic stabilization was accompanied by a substantially higher polar desolvation penalty (Δ*G*_polar_ = 62.04 kcal/mol), which partially offset the favorable electrostatic contribution. Collectively, these results suggest that Maculosin relies more heavily on electrostatic interactions for molecular recognition, whereas Gancidin W achieves binding stability predominantly through hydrophobic occupancy. Together, these factors likely contribute to the slightly more favorable binding energy observed for Gancidin W.

**Figure 8:**
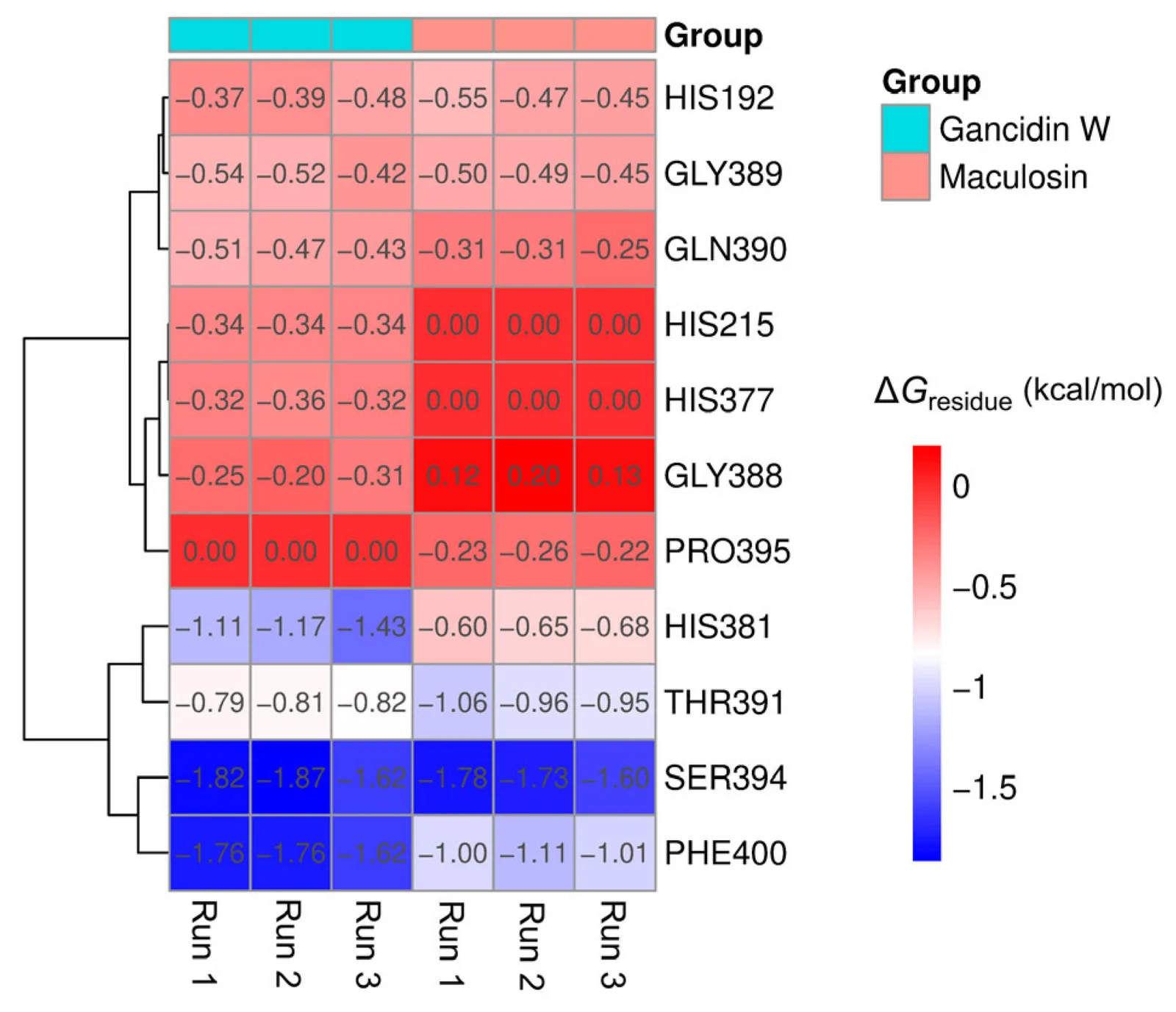
Hierarchical clustering heatmap of MM/GBSA per-residue energy contributions for Gancidin W and Maculosin. Residues were clustered using Ward’s minimum variance method (ward.D2) based on Euclidean distances.

To compare the residue interaction profiles of the two ligands, hierarchical clustering analysis was performed on the MM/GBSA per-residue energy contributions obtained from three independent MD trajectories (Figure 8). The heatmap revealed similar energy contribution patterns across replicate simulations, indicating that the residue-level energetic features were generally consistent among independent trajectories. SER394, PHE400, and THR391 exhibited persistent favorable contributions in both ligand-bound systems, whereas HIS215, HIS377, and HIS381 contributed more favorably to the Gancidin W complex. In contrast, PRO395 displayed a favorable contribution exclusively in the Maculosin complex. Overall, although the two ligands shared several favorable interaction residues, their residue energy contribution profiles differed considerably, suggesting differences in the local residue interactions contributing to ligand binding.

#### 2.3.4. Relative Binding Free Energy (RBFE) Analysis

**Figure 9:**
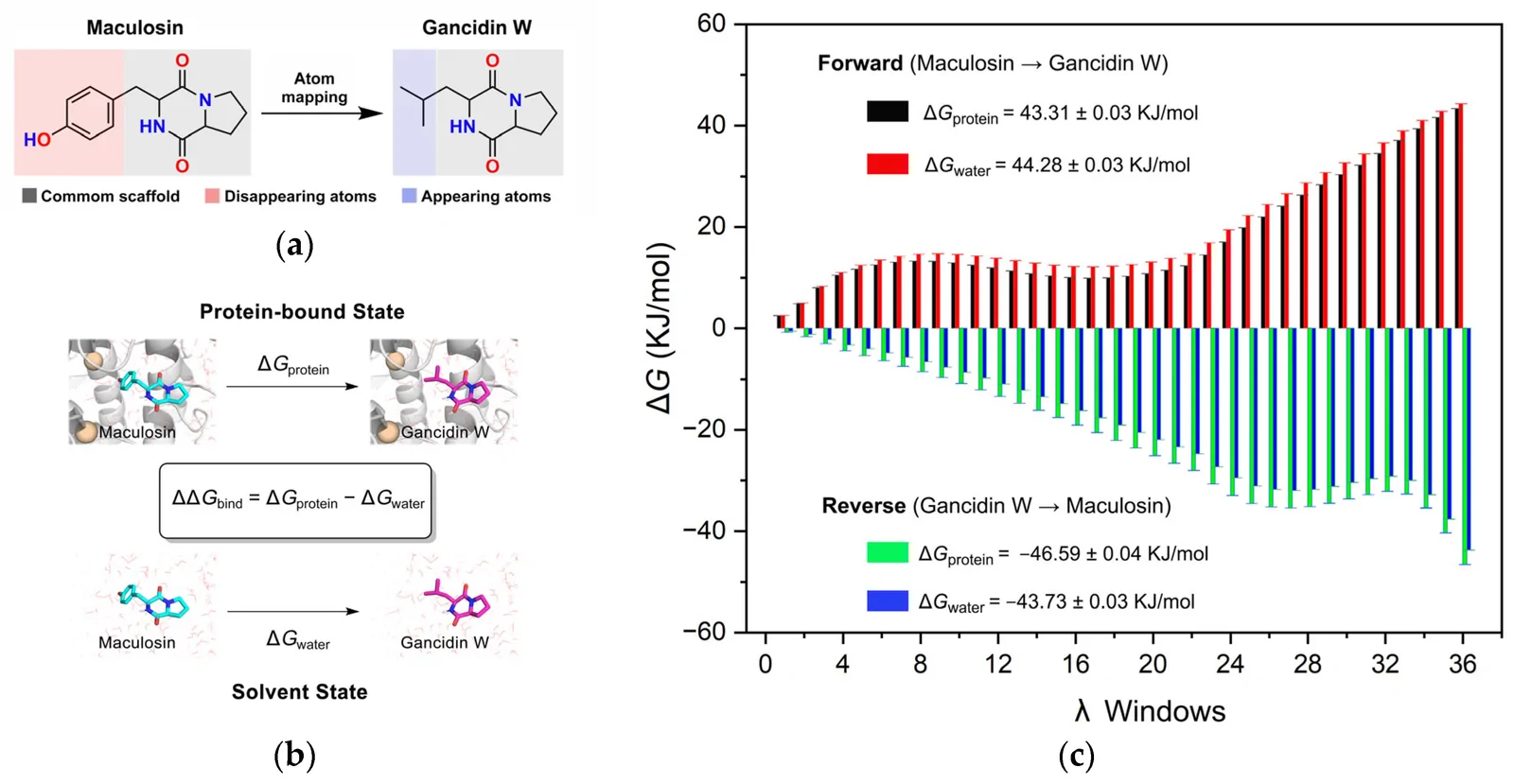
Construction and thermodynamic validation of the PMX-based RBFE workflow: (**a**) PMX-generated atom mapping between Maculosin and Gancidin W. (**b**) Alchemical thermodynamic cycle employed for RBFE calculations in the protein-bound and solvated states. (**c**) Cumulative free-energy profiles for forward (Maculosin → Gancidin W) and reverse (Gancidin W → Maculosin) alchemical transformations in the protein-bound and solvated states.

To further evaluate the RBFEs of Maculosin and Gancidin W, RBFE calculations were performed using an alchemical FEP approach. Prior to the RBFE simulations, the topological feasibility of the alchemical transformation between Maculosin and Gancidin W was assessed. PMX topology analysis successfully established the atom mapping between the shared scaffold and the transformed substituents of the two ligands (Figure 9a). The resulting dissimilarity score was 0.4545, which falls within the recommended threshold (<0.5) for reliable hybrid topology generation and subsequent RBFE calculations.

Based on the PMX-generated atom mapping, an alchemical thermodynamic cycle was constructed for RBFE calculations (Figure 9b). The binding process was decomposed into protein-bound and solvated thermodynamic branches. A unified coupling protocol was applied in both environments, and 37 non-uniformly distributed λ windows were employed for each alchemical transformation.

Relative free energies were estimated using the MBAR method, and cumulative free-energy profiles were obtained for both transformation directions (Figure 9c). For the forward transformation (Maculosin → Gancidin W), the cumulative free energies in the protein-bound and solvated states reached 43.31 and 44.28 kJ/mol, respectively. For the reverse transformation (Gancidin W → Maculosin), the corresponding values were −46.60 and −43.73 kJ/mol. Based on the thermodynamic cycle, the resulting RBFE differences (ΔΔ*G*_bind_) were −0.97 kJ/mol and −2.87 kJ/mol for the forward and reverse transformations, respectively.

Both independent alchemical pathways yielded negative ΔΔ*G* values, suggesting a thermodynamic preference for Gancidin W over Maculosin. Although minor hysteresis was observed, the calculated free-energy differences remained within a range supporting the convergence of the RBFE calculations. The averaged ΔΔ*G*_bind_ value of approximately −1.92 kJ/mol suggests a modest but favorable binding preference for Gancidin W. In addition, the RBFE predictions were qualitatively consistent with the experimentally observed activity trend in B16F10 cells, where Gancidin W exhibited greater tyrosinase inhibition than Maculosin (34.5% vs. 19.4%) [57], providing computational support for the observed ranking of activity. Additional quality-control analyses, including overlap matrix inspection and MBAR uncertainty estimates, supported the statistical reliability of the free-energy calculations (Figure S2).

## 3. Materials and Methods

### 3.1. Identification of Potential Targets Related to Gancidin W and Melanogenesis

The potential targets of Gancidin W were collected from Swiss Target Prediction (STP, http://www.swisstargetprediction.ch/, accessed on 11 March 2026) with a probability threshold of ≥0.30 [62] and were further supplemented using the Similarity Ensemble Approach database (SEA, https://sea.bkslab.org/, accessed on 11 March 2026) and the Super Pred database (https://prediction.charite.de/, accessed on 11 March 2026). Melanogenesis-related genes were retrieved from the Gene Cards Human Gene Database (Gene Cards, https://www.genecards.org/; relevance score ≥ 2 [62], accessed on 11 March 2026) and the Online Mendelian Inheritance in Man database (OMIM, https://www.omim.org/, accessed on 11 March 2026).

All the predicted targets were integrated, deduplicated, and standardized based on the official gene symbols of the HUGO Gene Nomenclature Committee (HGNC, https://www.genenames.org/, accessed on 11 March 2026). Non-protein-coding and redundant entries were excluded, and only unique human protein-coding genes were retained. The curated gene set was intersected with the Gancidin W target list using VENNY 2.1 (https://bioinfogp.cnb.csic.es/tools/venny/, accessed on 11 March 2026), and the overlapping targets were subjected to subsequent analyses.

### 3.2. Construction and Analysis of the Protein–Protein Interaction (PPI) Network

The overlapping targets were imported into the STRING database to construct a PPI network, with the organism restricted to *Homo sapiens* and the minimum required interaction score set to 0.4. For network visualization, only the interconnected nodes were retained, while isolated nodes and small disconnected subnetworks were excluded from the final network representation. The generated PPI network was subsequently visualized and analyzed using Cytoscape software (v3.9.1). Hub targets within the network were identified using the CytoHubba plugin based on degree centrality (DC) analysis, and the top 10 ranked targets were selected for subsequent analyses.

### 3.3. GO and KEGG Enrichment Analyses

The overlapping targets were submitted to the Database for Annotation, Visualization and Integrated Discovery (DAVID, https://davidbioinformatics.nih.gov, accessed on 12 March 2026) for Gene Ontology (GO) and Kyoto Encyclopedia of Genes and Genomes (KEGG) enrichment analyses. GO enrichment analysis included biological process (BP), cellular component (CC), and molecular function (MF) categories. GO terms and KEGG pathways were ranked according to statistical significance, and the top 10 enriched terms or pathways were selected for subsequent analyses. Terms and pathways with *p* < 0.05 and false discovery rate (FDR) < 0.05 were considered significantly enriched.

### 3.4. Molecular Docking Simulations Analysis

The crystal structures of CDK1 (PDB ID: 4Y72), CDK2 (PDB ID: 1H1R), PIK3CA (PDB ID: 4YKN), PIK3CD (PDB ID: 6G6W), MTOR (PDB ID: 6BCX), and STAT3 (PDB ID: 6NJS) were retrieved from the Protein Data Bank (PDB, https://www.rcsb.org/, accessed on 11 March 2026). Protein structures were prepared in PyMOL (version 2.5.5), while the structure of the ligand was obtained from PubChem and energy-minimized using OpenBabel (version 3.1.1) with the MMFF94 force field.

Molecular docking was performed using AutoDock Vina after protein and ligand preparation in AutoDock Tools (ADT) version 1.5.7. The docking grids were defined based on the co-crystallized ligand-binding sites. Docking reliability was validated by redocking the native ligands, yielding RMSD values below 2.0 Å. Protein–ligand interactions were subsequently analyzed using Discovery Studio 2019.

### 3.5. Cell-Based Evaluation of Anti-Melanogenic Activity

#### 3.5.1. Cell Culture and Viability Assay

B16F10 mouse melanoma cells (ATCC, Manassas, VA, USA) were maintained in DMEM (Thermo Fisher Scientific, Waltham, MA, USA) supplemented with 10% fetal bovine serum (Thermo Fisher Scientific, Waltham, MA, USA) and 1% penicillin–streptomycin (Thermo Fisher Scientific, Waltham, MA, USA) at 37 °C in a humidified atmosphere containing 5% CO_2_. Cell viability was assessed using an MTT assay. Cells were seeded in 24-well plates (1.5 × 10^4^ cells/well) and pre-incubated for 24 h prior to treatment with Gancidin W for 72 h. MTT solution (0.2 mg/mL; Sigma-Aldrich, St. Louis, MO, USA) was added and incubated for 4 h at 37 °C. Formazan crystals were dissolved in DMSO (Biosesang, Seongnam, Republic of Korea), and absorbance was measured at 540 nm using a microplate reader.

#### 3.5.2. Melanin Content Assay

Cells were seeded in 60 mm dishes (8.0 × 10^4^ cells/dish) and treated with the test compound in the presence of 100 nM α-MSH (Sigma-Aldrich, St. Louis, MO, USA) for 72 h. Cells were washed with ice-cold PBS (Biosesang, Seongnam, Republic of Korea) and lysed in RIPA buffer (Biosesang, Seongnam, Republic of Korea) containing a protease inhibitor cocktail (1%; Sigma-Aldrich, St. Louis, MO, USA) at 4 °C for 30 min. Lysates were centrifuged at 15,000 rpm for 30 min at 4 °C, and pellets were dissolved in 1 N NaOH (Sigma-Aldrich, St. Louis, MO, USA) containing 10% DMSO at 80 °C for 20 min. Melanin content was quantified by measuring absorbance at 405 nm.

#### 3.5.3. Intracellular Tyrosinase Activity Assay

Cells were seeded in 60 mm dishes (8.0 × 10^4^ cells/dish) and treated under identical experimental conditions as the melanin content assay. Cells were lysed in RIPA buffer and centrifuged at 15,000 rpm for 30 min at 4 °C. Protein concentration was determined using a BCA assay (Thermo Fisher Scientific, Waltham, MA, USA) after 1:10 dilution of supernatants. Tyrosinase activity was measured by incubating 20 µL of protein extract with 80 µL of L-DOPA (2 mg/mL in 0.1 M sodium phosphate buffer, pH 6.8; Sigma-Aldrich, St. Louis, MO, USA). Absorbance at 490 nm was monitored kinetically every 30 min for 60 min at 37 °C.

### 3.6. Statistical Analysis

Data are presented as the mean ± standard deviation (SD) of three independent experiments. Statistical analyses were performed using GraphPad Prism software (version 10.0; GraphPad Software, San Diego, CA, USA). Statistical significance among groups was determined by one-way analysis of variance (ANOVA) followed by Tukey’s multiple comparisons test. Differences were considered statistically significant at *p* < 0.05.

### 3.7. Molecular Dynamics (MD) Simulations Analysis

MD simulations were performed using GROMACS 2024.4. Protein structures were modeled using the AMBER14SB force field. Ligand parameters were generated using the GAFF2 force field, and AM1-BCC atomic charges were assigned through ACPYPE. All systems were solvated in explicit TIP3P water and neutralized by adding Na^+^ and Cl^−^ ions to achieve a physiological salt concentration of 0.15 M. A cubic simulation box was constructed with a minimum solute-boundary distance of 10 Å under periodic boundary conditions (PBC).

Following initial energy minimization, a unified equilibration and production pipeline was applied to all the simulation systems across this study. The equilibration protocol consisted of a canonical (NVT) step (500 ps, 310 K, V-rescale thermostat) followed by an isothermal-isobaric (NPT) step (500 ps, 1 bar, Parrinello–Rahman barostat) [63,64]. Long-range electrostatic interactions were treated using the particle mesh Ewald (PME) method with a 1.2 nm real-space cutoff, while van der Waals interactions were treated with a 1.2 nm cutoff. To enable an integration time step of 2 fs, all covalent bonds involving hydrogen atoms were constrained using the LINCS algorithm [65].

End-state binding free energies were calculated using gmx_MMPBSA from conformations extracted from the final 20 ns of each production trajectory. The GB-OBC2 implicit solvent model (igb = 5) with a salt concentration of 0.150 M was employed. Per-residue free-energy decomposition was performed for residues within 4 Å of the ligand [66]. Entropic contributions were not included.

#### 3.7.1. MD Simulations for Network Pharmacology Targets

Each system was first subjected to energy minimization using the steepest descent algorithm, followed by equilibration as described in Section 3.7. Subsequently, 100 ns production MD simulations were performed under NPT conditions starting from the equilibrated structures. The generated trajectories were used for subsequent analyses of structural stability and protein–ligand interactions.

#### 3.7.2. MD Simulations of hTYRP1–Ligand Complexes

Building upon our previous work [43,57], in which the molecular docking of Gancidin W and Maculosin with hTYRP1 was reported, the resulting docking poses were used as the initial binding conformations for MD simulations. The simulation protocol was further optimized to improve sampling reproducibility and to ensure the accuracy required for subsequent FEP calculations. MD simulations were conducted for the hTYRP1–ligand complexes using the hTYRP1 crystal structure (PDB ID: 5M8M; co-crystallized with kojic acid) to investigate their dynamic stability and protein–ligand interaction characteristics. The initial complexes were energy-minimized using a two-step protocol (steepest descent followed by L-BFGS) [67], followed by equilibration as described in Section 3.7. To evaluate simulation reproducibility, three independent replicas were generated for each complex using different random velocity seeds during NVT equilibration and subsequently propagated for 100 ns production simulations.

Protein–ligand interactions were analyzed using MDanalysis (version 2.9.0). To evaluate metal–ligand binding stability, the minimum atomic distance across all the trajectories was analyzed via Kernel Density Estimation (KDE). Interaction occupancies were evaluated over the entire MD trajectory as time-averaged frame fractions. Interactions were identified using geometric criteria: hydrogen bonds (distance ≤ 3.5 Å, angle ≥ 120°), hydrophobic contacts (≤4.5 Å), salt bridges (≤4.0 Å), and π–π interactions (centroid distance ≤ 5.0 Å, ring angle ≤ 30°). The resulting occupancies were encoded into protein–ligand interaction fingerprints (PLIF) to quantify interaction persistence throughout the simulations.

#### 3.7.3. Alchemical Free Energy Perturbation (FEP) Calculations for hTYRP1–Ligand Complexes

Relative binding free energies (RBFEs, ΔΔ*G*_bind_) were estimated using FEP calculations for the hTYRP1 systems. Hybrid topologies and coordinates for ligand transformations were generated using PMX [68].

For both the ligand-in-water and protein-bound states, alchemical transformations were performed using 37 λ windows spanning the interval from 0.00 to 1.00. Electrostatic and van der Waals interactions were simultaneously scaled along the λ pathway. To alleviate endpoint singularities and numerical instabilities during alchemical transformations, soft-core potentials were applied to non-bonded interactions using sc-alpha = 0.5, sc-sigma = 0.3 nm, and sc-power = 1 [69].

Each λ window was first energy-minimized as described in Section 3.7.2, followed by equilibration according to the protocol described in Section 3.7. Production simulations were subsequently performed for 5 ns per λ window under NPT conditions. Relative free energy differences were calculated using the Multistate Bennett Acceptance Ratio (MBAR) estimator implemented in the alchemlyb package (version 2.3.0) [70].

### 3.8. Density Functional Theory (DFT) Calculations

All the DFT calculations were performed using Gaussian 16. Geometries were optimized at the B3LYP/6-311+G(d,p) level [71,72], and the absence of imaginary frequencies confirmed all the structures as true local minima on the potential energy surface. Molecular electrostatic potential (MEP) surfaces and frontier molecular orbitals (FMOs) were visualized and analyzed using GaussView 6.0.

## 4. Conclusions

Building upon our previous identification of Gancidin W as an mTYR inhibitor, this study further substantiates the anti-melanogenic efficacy of this ecologically prevalent and dietary-accessible cyclic dipeptide for the first time in α-MSH-stimulated B16F10 melanoma cells. Through the integration of cellular evaluation and multiscale computational analyses, this work provides a comprehensive characterization of the interaction properties of Gancidin W with the melanogenesis-related target hTYRP1, and reveals distinct molecular recognition features relative to the structurally related diketopiperazine Maculosin, and further provides the first RBFE/FEP-based relative binding free energy evaluation for the hTYRP1 system. These findings broaden the biological profile of Gancidin W and highlight the potential of diketopiperazine natural products as a valuable source for the discovery of novel depigmenting agents.

## Figures and Tables

**Table 1:** Comparison of MM/GBSA binding free energies (kcal/mol) for Gancidin W and Maculosin.

Component	Gancidin W (Mean ± SD)	Maculosin (Mean ± SD)
Δ*E*_vdW_	−32.51 ± 0.89	−28.11 ± 0.38
Δ*E*_ele_	−6.06 ± 0.06	−54.44 ± 0.83
Δ*G*_polar_	13.51 ± 0.18	62.04 ± 0.65
Δ*G*_nonpolar_	−3.70 ± 0.02	−3.81 ± 0.04
Δ*E*_gas_	−38.57 ± 0.90	−82.55 ± 1.15
Δ*G*_solv_	9.80 ± 0.17	58.23 ± 1.12
Δ*G*_bind_	−28.77 ± 0.75	−24.32 ± 0.57

## Data Availability

All data generated or analyzed during this study are fully available within this published article.

## Supplementary Materials

The following supporting information can be downloaded at https://www.mdpi.com/article/10.3390/ph19081191/s1, Table S1: Predicted targets of Gancidin W, hyperpigmentation-related targets, and common targets; Table S2: Binding free energy analysis of the CDK1-Gancidin W complex; Table S3: Binding free energy analysis of the CDK2-Gancidin W complex; Table S4: Binding free energy analysis of the PIK3CA-Gancidin W complex; Table S5: Residue energy decomposition analysis of the CDK1-Gancidin W complex; Table S6: Residue energy decomposition analysis of the CDK2-Gancidin W complex; Table S7: Residue energy decomposition analysis of the PIK3CA-Gancidin W complex; Table S8: Detailed MM/GBSA energy components for Gancidin W and Maculosin across triplicate simulations; Figure S1: MD simulation analyses of Gancidin W–protein complexes; Figure S2: Overlap matrix analysis for forward and reverse alchemical transformations in the protein-bound and solvated states.
