# Supplementary material for: Computational and Cellular Evaluation of the Anti-Melanogenic Potential of Gancidin W: Integrating Network Pharmacology and Free-Energy Analyses
Source: Pharmaceuticals (Basel). 2026 Jul 29;19(8):1191. doi: 10.3390/ph19081191 (PMC13516670; doi:10.3390/ph19081191)
Supplement: Supplementary file 1 [file pharmaceuticals-19-01191-s001.zip › pharmaceuticals-4426321-supplementary.pdf]

Computational and Cellular Evaluation of the Anti-Melanogenic Potential of Gancidin W: Integrating Network Pharmacology and Free-Energy Analyses

Yang Xu, and Chang-Gu Hyun*

Jeju Inside Agency and Cosmetic Science Center, Department of Chemistry and Cosmetics, Jeju National University, Jeju 63243, Republic of Korea

* Correspondence: cghyun@jejunu.ac.kr; Tel.: +82-64-754-1803

CONTENTS

Table S1. Predicted targets of Gancidin W, hyperpigmentation-related targets, and common targets. 2

Table S2. Binding free energy analysis of the CDK1- Gancidin W complex. 4

Table S3. Binding free energy analysis of the CDK2- Gancidin W complex. 4

Table S4. Binding free energy analysis of the PIK3CA - Gancidin W complex. 4

Table S5. Residue energy decomposition analysis of the CDK1- Gancidin W complex. 4

Table S6. Residue energy decomposition analysis of the CDK2- Gancidin W complex. 4

Table S7. Residue energy decomposition analysis of the PIK3CA - Gancidin W complex. 5

Table S8. Detailed MM/GBSA energy components for Gancidin W and Maculosin across triplicate simulations. 5

Figure S1. MD simulation analyses of Gancidin W–protein complexes. 5

Figure S2. Overlap matrix analysis for forward and reverse alchemical transformations in the protein-bound and solvated states. 6

Table S1. Predicted targets of Gancidin W, hyperpigmentation-related targets, and common targets.

Category	symbols
Potential targets of Gancidin W (260)	AAK1, ABCG2, ABCC1, ACACA, ACACB, ACE, ACKR3, ADAM10, ADAM8, ADORA1, ADORA2B, ADRB1, ANPEP, APEX1, APLNR, AR, ATAD2, AURKB, AVPR1A, AVPR2, AXL, BACE1, BIRC2, BIRC3, BLM, BMP2K, BRD4, CACNA1B, CACNA1H, CAPN1, CAPNS1, CARM1, CASP7, CA7, CCNB1, CCNB2, CCNB3, CCND1, CCNE1, CCNE2, CCR1, CCR5, CD38, CDK1, CDK2, CDK4, CDK5, CDK5R1, CDC25A, CDC25B, CDC25C, CELA1, CFTR, CHRNA3, CHRNA4, CHRNB2, CHRNB4, CHUK, CLK4, CNR2, CSNK2A1, CSNK2B, CTSB, CTSC, CTSD, CTSH, CTSK, CTSL, CTSS, CYP11B2, CYP19A1, CYP3A4, CYSLTR2, DCUN1D1, DPP4, DPP7, DPP8, DPP9, DUSP3, DUT, EDNRA, EDNRB, EGLN1, ELANE, ENPP2, EPHA2, EPHX1, ESR2, EZH2, F10, F2, F7, FABP4, FAP, FCGRT, FKBP1A, FPR1, FPR2, FURIN, GABRA1, GABRA2, GABRA3, GABRA5, GABRA6, GABRB2, GABRB3, GABRG2, GALR1, GALR2, GHSR, GLRA1, GPBAR1, GPR35, GPR55, GPR139, GRB2, GRIN1, GRIN2B, GUSB, GZMB, HCRTR1, HCRTR2, HDAC1, HDAC10, HDAC11, HDAC2, HDAC3, HDAC4, HDAC5, HDAC6, HDAC8, HGFAC, HIF1A, HLA-A, HMOX1, HPN, HRH1, HRH3, HSD11B1, HSD11B2, HTR2C, HTR7, IDO1, ITGA5, ITGB1, ITK, JAK3, KEAP1, KDM1A, KDM4A, KDM4C, KIF11, KISS1R, KLF5, KLK7, KLKB1, LAP3, LDHA, LNPEP, MAOB, MC1R, MC3R, MC4R, MEN1, MGLL, MMP1, MMP2, MMP7, MMP8, MMP9, MMP14, MTNR1A, MTNR1B, MTOR, NAAA, NAMPT, NCOR2, NFKB1, NOS2, NOS3, NR1I2, NR3C2, NRP1, NTRK3, NTSR2, NT5E, NQO2, OPRD1, OPRM1, OXTR, P2RX7, PARP1, PDE3B, PDE4B, PDE7A, PDGFRA, PDGFRB, PEPD, PHF8, PIK3CA, PIK3CD, PIK3R1, PIN1, PKN1, PPIA, PRKAA1, PRKCD, PREP, PRSS1, PSEN1, PSMB5, PSMB9, PTGS1, PTGS2, PTK2B, PTPN1, PTPN2, QRFPR, RCOR1, RORC, RPS6KA1, RPS6KA2, SCD, SCN2A, SCN3A, SCN4A, SCN9A, SIGMAR1, SIRT1, SIRT2, SIRT3, SLC2A1, SLC40A1, SLC5A1, SLC6A2, SLC6A3, SLC6A4, SLC9A1, SOAT1, SPHK1, ST14, STAT3, TAAR1, TACR1, TDO2, TDP1, THRA, TLR4, TMEM173, TMPRSS6, TNFRSF10A, TNKS2, TOP2A, TRHR, TRIM24, TLR4, TYMP, TYRO3, WDR5, XIAP, ZAP70
Hyperpigmentation-related targets (440)	ABCA1, ABCA12, ABCA3, ABCA4, ABCB1, ABCB10, ABCB11, ABCB4, ABCB5, ABCB6, ABCB7, ABCB8, ABCC1, ABCC2, ABCC3, ABCC4, ABCC6, ABCD1, ABCD4, ABL1, ABL2, ACE, ACE2, ACVR1, ACVR2A, ADAM10, ADAM17, ADAM9, ADAMTS10, ADAMTS13, ADAMTS17, ADIPOQ, ADRB1, ADRB2, AGT, AGTR1, AHR, AKT1, AKT2, AKT3, ALB, ALK, ALOX15, ANGPT1, ANGPT2, ANXA5, APC, AR, ARAF, ARID1A, ARNT, ASIP, ATM, ATR, ATRX, AURKA, AURKB, AXL, AXIN1, AXIN2, B2M, BACE2, BAP1, BARD1, BAX, BCL10, BCL11A, BCL2, BCL2L1, BCL2L11, BIRC5, BLM, BMPR1A, BMPR2, BRAF, BRCA1, BRCA2, BTK, BUB1, BUB1B,

	CASP1, CASP3, CASP8, CASP9, CAT, CBL, CCL2, CCL3, CCL4, CCL5, CCNA2, CCNB1, CCND1, CCND3, CCNE1, CD14, CD28, CD34, CD36, CD38, CD40LG, CD44, CD68, CD79A, CD86, CDC20, CDC25A, CDC25C, CDK1, CDK2, CDK4, CDK6, CDK7, CDKN1A, CDKN1B, CDKN1C, CDKN2A, CDKN2B, CDKN2C, CEBPA, CFTR, CHEK1, CHEK2, CHUK, CREB1, CREBBP, CRH, CRHR1, CSF1, CSF2, CSF3, CSF3R, CTNNB1, CXCL1, CXCL10, CXCL12, CXCL8, CXCR2, CXCR3, CXCR4, CYCS, CYP11A1, CYP11B1, CYP11B2, CYP17A1, CYP19A1, CYP1A2, CYP2C19, CYP2C9, CYP2D6, CYP3A4, DCT, DDR1, DDR2, DICER1, DKK1, DKK4, DNMT1, DNMT3A, DNMT3B, DRD4, EDN1, EDN3, EDNRA, EDNRB, EGF, EGFR, ELANE, ENO1, EP300, EPAS1, EPHA2, EPHA3, EPHA4, ERBB2, ERBB3, ERBB4, ESR1, ESRRB, ETV1, ETV4, EZH2, F2, F3, F9, FADD, FANCA, FANCC, FANCD2, FANCE, FANCF, FANCG, FANCI, FANCL, FANCM, FAS, FASLG, FGF1, FGF10, FGF13, FGF16, FGF17, FGF18, FGF19, FGF2, FGF20, FGF22, FGF3, FGF4, FGF5, FGF6, FGF7, FGF8, FGF9, FGFR1, FGFR2, FGFR3, FGFR4, FLT1, FLT3, FLT4, FOS, FOXO1, FOXO3, FOXP3, FURIN, GATA1, GATA2, GATA3, GATA4, GCH1, GHR, GHRHR, GHRL, GLI1, GLI2, GLI3, GNAS, GSK3B, GTF2I, HAMP, HBEGF, HGF, HIF1A, HLA-A, HLA-B, HLA-C, HLA-DQB1, HMOX1, HRAS, HSP90AA1, HSP90AB1, HSPA5, HSPA8, HSPA9, HTR1A, HTR2C, IDH1, IDO1, IFNG, IGF1, IGF1R, IL10, IL13, IL15, IL17A, IL18, IL1A, IL1B, IL2, IL3, IL4, IL5, IL6, IL7, INSR, IRF1, IRF3, IRF4, IRF5, ITGA2B, ITGA4, ITGA5, ITGA6, ITGB1, ITGB2, ITGB4, JAK2, JAK3, JUN, KDR, KIT, KITLG, KRAS, LCK, LEF1, LRRK2, MAP2K1, MAP2K2, MAPK1, MAPK10, MAPK14, MAPK3, MAPK8, MC1R, MCL1, MDM2, MDM4, MET, MITF, MKI67, MLH1, MMP1, MMP14, MMP2, MMP7, MMP9, MTOR, MYC, MYCN, MYD88, NF1, NFKB1, NFKBIA, NOS1, NOS2, NOS3, NR3C1, NR3C2, NRAS, NRP1, NTRK1, NTRK2, NTRK3, PDGFRA, PDGFRB, PDGFA, PDGFB, PIK3CA, PIK3CB, PIK3CD, PIK3CG, PIK3R1, PLCG1, PPARG, PRKACA, PRKCA, PRKCB, PRKCD, PRKCQ, PRKCZ, PTEN, PTK2, PTPN11, PTPN22, PTPN6, RAF1, RAC1, RHO, RHOA, RB1, RELA, RET, RIPK1, RIPK2, RPS6KA1, RPS6KB1, RUNX1, RUNX3, SASH1, SDHA, SDHB, SDHC, SDHD, SERPINA1, SHH, SIRT1, SMAD4, SMO, SOCS3, SOX10, SOX9, SRC, STAT1, STAT2, STAT3, STAT4, STAT5A, STAT5B, STAT6, SREBF1, STK11, SYK, TAB2, TBP, TGFB1, TGFB2, TGFB3, TGFBR2, TLR2, TLR3, TLR4, TLR7, TLR8, TLR9, TNF, TNFRSF10A, TNFRSF1A, TNFRSF1B, TOP1, TOP2A, TP53, TP73, TRAF6, TSHR, TSC1, TSC2, TYR, TYRP1, ULK1, VEGFA, VEGFB, VEGFC, VEGFD, VHL, WAS, WEE1, WIF1, WNT1, WNT3, WNT3A, WNT4, WNT5A, WNT6, WNT9A, WWOX, XIAP, YAP1, ZEB1, ZEB2
Common targets (63)	ABCC1, ACE, ADAM10, ADRB1, AR, AURKB, AXL, BLM, CCNB1, CCND1, CCNE1, CD38, CDK1, CDK2, CDK4, CDC25A, CDC25C, CFTR, CHUK, CYP11B2, CYP19A1, CYP3A4, EDNRA, EDNRB, ELANE, EPHA2, EZH2, F2, FURIN, HIF1A, HLA-A, HMOX1, HTR2C, IDO1, ITGA5, ITGB1, JAK3, MC1R, MMP1, MMP2, MMP7, MMP9, MMP14, MTOR, NFKB1, NOS2, NOS3,

	NR3C2, NRP1, NTRK3, PDGFRA, PDGFRB, PIK3CA, PIK3CD, PIK3R1, PRKCD, RPS6KA1, SIRT1, STAT3, TLR4, TNFRSF10A, TOP2A, XIAP

Table S2. Binding free energy analysis of the CDK1–Gancidin W complex.

Frames	VDWAALS	EEL	EGB	ESURF	GGAS	GSOLV	TOTAL
Average	-31.73	-1.66	8.93	-3.93	-33.38	5	-28.39
SD	3.21	2.51	2.07	0.28	2.71	2.2	2.96
SEM	0.7	0.55	0.45	0.06	0.59	0.48	0.65

Table S3. Binding free energy analysis of the CDK2–Gancidin W complex.

Frames	VDWAALS	EEL	EGB	ESURF	GGAS	GSOLV	TOTAL
Average	-35.18	-2.69	11.34	-4.21	-37.88	7.13	-30.75
SD	2.47	0.72	0.85	0.07	2.45	0.85	2.45
SEM	0.54	0.16	0.19	0.01	0.54	0.19	0.54

Table S4. Binding free energy analysis of the PIK3CA–Gancidin W complex.

Frames	VDWAALS	EEL	EGB	ESURF	GGAS	GSOLV	TOTAL
Average	-34.35	-5.37	8.95	-4.04	-39.72	4.92	-34.8
SD	1.87	1.11	0.79	0.12	2.24	0.79	1.84
SEM	0.41	0.24	0.17	0.03	0.49	0.17	0.4

Table S5. Residue energy decomposition analysis of the CDK1–Gancidin W complex.

Frames	ILE:10	VAL:18	ALA:31	PHE:80	PHE:82	LEU:83	MET:85	ASP:86	LEU:135
Average	-1.94	-1.2	-0.77	-0.98	-0.79	-0.82	-0.52	-0.77	-2.05
SD	0.5	0.42	0.2	0.35	0.38	0.47	0.22	0.3	0.79
SEM	0.11	0.09	0.04	0.08	0.08	0.1	0.05	0.07	0.17

Table S6. Residue energy decomposition analysis of the CDK2–Gancidin W complex.

Frames	ILE: 10	GLY: 11	GLU: 12	GLY: 13	VAL: 18	VAL: 64	PHE: 80	ASP: 86	ASN: 132	LEU: 134	ALA: 144	ASP: 145
Average	-1.08	-0.62	-0.85	-0.77	-1.84	-0.55	-1.05	-0.55	-0.7	-2.14	-0.63	-1.05
SD	0.42	0.4	0.32	0.27	0.55	0.19	0.31	0.24	0.22	0.33	0.15	0.3
SEM	0.09	0.09	0.07	0.06	0.12	0.04	0.07	0.05	0.05	0.07	0.03	0.06

Table S7. Residue energy decomposition analysis of the PIK3CA–Gancidin W complex.

Frames	MET: 1772	TRP: 1780	ILE: 1800	LEU: 1834	TYR: 1836	ILE: 1848	GLU: 1849	VAL: 1850	VAL: 1851	MET: 1922	PHE: 1930	ILE: 1932
Average	-0.56	-0.87	-0.97	-0.51	-2.77	-1	-0.56	-1.91	-1.27	-1.26	-1.41	-1.87
SD	0.21	0.16	0.46	0.16	0.45	0.11	0.27	0.28	0.33	0.26	0.23	0.35
SEM	0.05	0.04	0.1	0.03	0.1	0.02	0.06	0.06	0.07	0.06	0.05	0.08

Table S8. Detailed MM/GBSA energy components for Gancidin W and Maculosin across triplicate simulations. (kcal/mol).

Ligand	Component	Run 1	Run 2	Run 3	Average ± SD
Gancidin W	VDWAALS	-33.11	-32.94	-31.49	-32.51 ± 0.89
	EEL	-6.12	-6.01	-6.06	-6.06 ± 0.06
	EGB	13.66	13.56	13.30	13.51 ± 0.18
	ESURF	-3.70	-3.72	-3.69	-3.70 ± 0.02
	GGAS	-39.22	-38.95	-37.55	-38.57 ± 0.90
	GSOLV	9.96	9.83	9.62	9.80 ± 0.17
	TOTAL	-29.27	-29.12	-27.93	-28.77 ± 0.75
Maculosin	VDWAALS	-28.53	-27.81	-27.99	-28.11 ± 0.38
	EEL	-55.39	-53.96	-53.98	-54.44 ± 0.83
	EGB	62.79	61.52	61.81	62.04 ± 0.65
	ESURF	-3.85	-3.80	-3.78	-3.81 ± 0.04
	GGAS	-83.92	-81.77	-81.96	-82.55 ± 1.15
	GSOLV	58.94	57.73	58.03	58.23 ± 1.12
	TOTAL	-24.98	-24.05	-23.93	-24.32 ± 0.57

Figure S1. MD simulation analyses of Gancidin W–protein complexes.

(**a**) RMSD profiles. RMSF profiles of (**b**) CDK1, (**c**) CDK2, and (**d**) PIK3CA complexes. Rg analyses of (**e**) CDK1 and CDK2 complexes and (**f**) PIK3CA complexes. SASA analyses of (**g**) CDK1 and CDK2 complexes and (**h**) PIK3CA complexes. Hydrogen bond analyses of (**i**) CDK1, (**j**) CDK2, and (**k**) PIK3CA complexes.

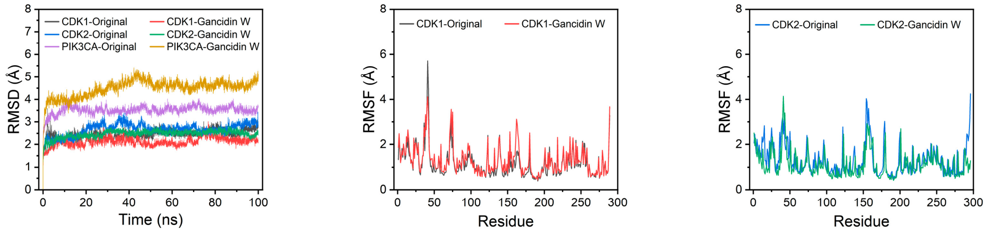

(**a**) (**b**) (**c**)

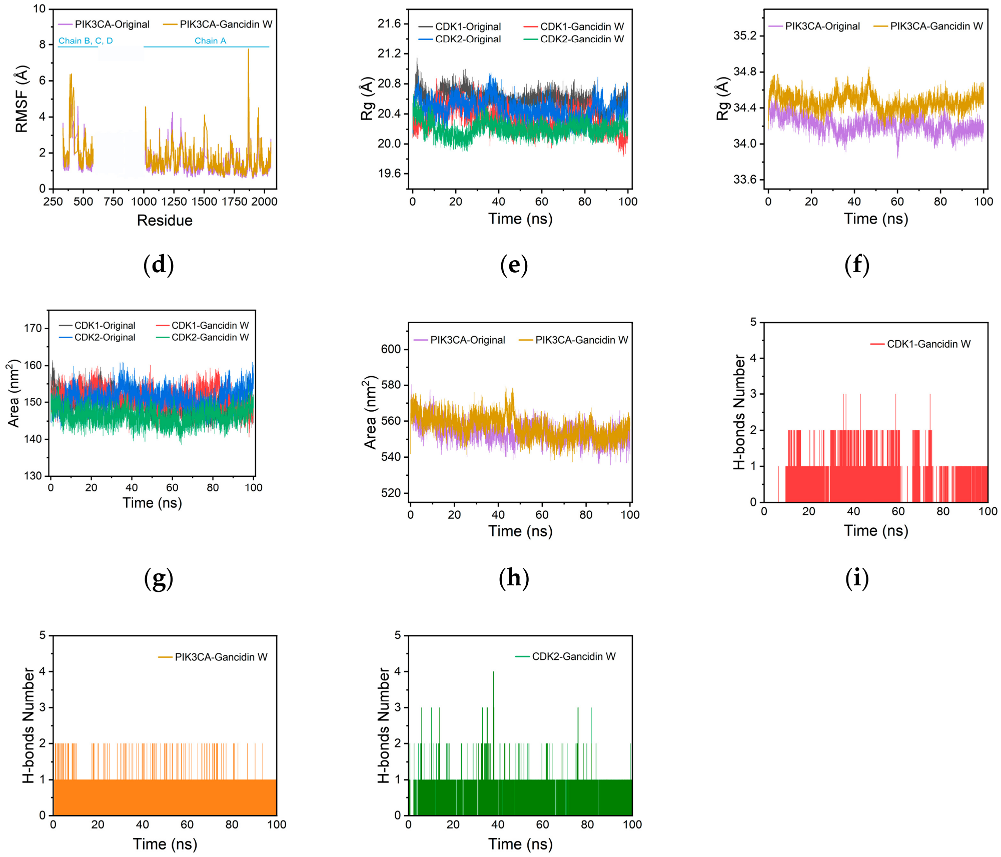

(**d**) (**e**) (**f**)

(**g**) (**h**) (**i**)

(**j**) (**k**)

Figure S2. Overlap matrix analysis for forward and reverse alchemical transformations in the protein-bound and solvated states.

(**a**) Forward, protein-bound; (**b**) forward, solvated; (**c**) reverse, protein-bound; (**d**) reverse, solvated.

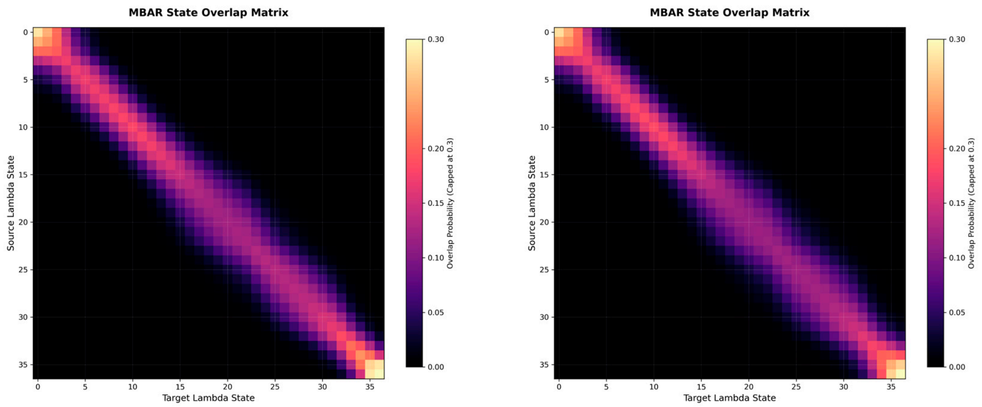

(**a**) (**b**)

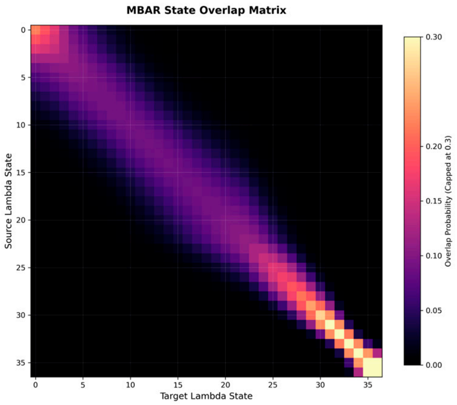
MBAR State Overlap Matrix
Source Lambda State
Target Lambda State
Overlap Probability (Capped at 0.3)

(c)

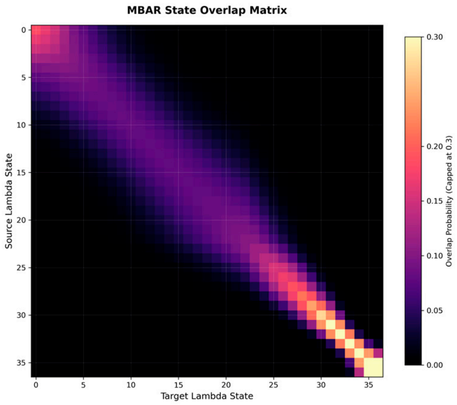
MBAR State Overlap Matrix
Source Lambda State
Target Lambda State
Overlap Probability (Capped at 0.3)

(d)
